# Whole-Genome Analysis of Termite-Derived *Bacillus velezensis* BV-10 and Its Application in King Grass Silage

**DOI:** 10.3390/microorganisms11112697

**Published:** 2023-11-03

**Authors:** Xingbo Zhang, Xiaotao He, Jieru Chen, Jingtao Li, Yuhui Wu, Yu Chen, Yuhui Yang

**Affiliations:** College of Tropical Agriculture and Forestry, Hainan University, Haikou 570228, China; 15105690350@163.com (X.Z.); 18892990995@163.com (X.H.); 15501877508@163.com (J.C.); l15839127638@163.com (J.L.); 18417266830@163.com (Y.W.); ychen@hainanu.edu.cn (Y.C.)

**Keywords:** *Bacillus velezensis* BV-10, whole-genome sequencing, king grass silage, cellulose-degrading

## Abstract

*Bacillus velezensis* (*B*. *velezensis*) is a cellulose-degrading strain that has the potential as an additive in fermented feed. *B*. *velezensis* BV-10 was isolated and screened from the termite gut. We sequenced the whole genome of this new source of *B*. *velezensis* to reveal its potential for use in cellulose degradation. Whole-genome sequencing of *B. velezensis* BV-10 showed that it has a circular chromosome of 3929792 bp containing 3873 coding genes with a GC content of 45.51% and many genes related to cellulose, hemicellulose, and lignin degradation. King grass silage was inoculated with *B. velezensis* BV-10 and mixed with other feed additives to assess the effect of *B. velezensis* BV-10 on the fermentation quality of silage. Six treatment groups were established: the control, *B. velezensis* BV-10, molasses, cellulase, *B. velezensis* BV-10 plus molasses, and *B. velezensis* BV-10 plus cellulase groups. After 30 days of silage-fermentation testing, *B. velezensis* BV-10 was found to rapidly reduce the silage pH value and significantly reduce the acid-detergent fiber (ADF) content (*p* < 0.05). The addition of *B. velezensis* BV-10 plus molasses and cellulase in fermented feed significantly reduced the silage neutral-detergent fiber and ADF content and promoted organic-acid accumulation (*p* < 0.05). The above results demonstrate that *B. velezensis* BV-10 promotes the fermentation quality of silage and that this effect is greater when other silage-fermentation additives are included. In conclusion, genes involved in cellulose degradation in *B. velezensis* BV-10 were identified by whole-genome sequencing and further experiments explored the effects of *B. velezensis* BV-10 and different feed additives on the fermentation quality of king grass silage, revealing the potential of *Bacillus velezensis* as a new silage additive.

## 1. Introduction

King grass (*Pennisetum purpureum Rich* × *Pennisetum americanum*) can be planted in tropical and subtropical areas; with its high yield and rich nutritional value, king grass is the main feed resource for ruminants in such regions [1]. The growth of king grass is seasonal, with strong growth in the summer or rainy seasons and slow growth in the winter or dry seasons, leading to an imbalance in the supply and demand of king grass; thus, suitable preservation methods are required to provide a continuous feed supply for ruminants [2]. In Hainan, silage technology is mainly used to preserve king grass. Silage is a long-term forage-storing method and improves palatability by compacting and sealing the forage and preventing spoilage through fermentation by lactic acid bacteria, which reduces the pH value of the forage and inhibits the growth of harmful bacteria. Many studies have shown that the addition of molasses [3], acids [4], enzymes [5] and microbial additives [6] to silage can improve its fermentation quality and efficiency. The predominant enzyme treatment is cellulase, a complex enzyme system comprising multiple hydrolytic enzymes that can decompose cellulose into oligosaccharides and monosaccharides [7]. Cellulase can be added to silage to promote cellulose degradation and increase the content of substrates for silage fermentation, improving silage fermentation quality. Currently, a large number of cellulases are produced by fungi, but bacterial cellulases have advantages over fungal cellulases with regard to facilitated mass transfer, increased specific activity and improved stability [8]. Bacterial cellulases are extracted from the genera *Bacillus* sp. [9], *Fusobacterium* sp. [10], *Fibrosporum* sp. [11] and *Clostridium* sp. [12]. Microbial additives are better than enzymes for lignocellulosic hydrolysis pretreatment [13]. Microbial additives mainly include *Lactobacillus* sp., *Saccharomyces cerevisiae* sp., and cellulase-producing *Bacillus* sp. Zhang et al. [14] added *Bacillus subtilis* to maize silage and found that these microbes could reduce the cellulose content in the feed and improve the silage quality.

Termites are a model organism for lignocellulosic biomass pretreatment under natural conditions [15] and can remove 74–99% of cellulose and 65–87% of hemicellulose from wood samples [16]. The degradation level of wheat straw by microflora in the gut of termites is as high as 45% [17]. In recent years, many cellulose-degrading bacteria have been identified from termites; Bahiru et al. [18] isolated a strain of *Bacillus* sp., bacterium with cellulose- and hemicellulose-degrading abilities from wood-feeding termites and applied it to rice straw, with good results. At least one bacterial species of the genus *Bacillus*, *Bacillus velezensis* (*B. velezensis*), can degrade crude fiber, degrade cellulose into reducing sugars, increase soluble carbohydrates, and improve the fermentation substrate content [19]. *B. velezensis* may not be able to directly hydrolyze cellobiose because it lacks exocellulase, limiting its cellulose-degrading activity; thus, it is necessary to add appropriate exogenous enzymes for synergistic treatment [20]. To the best of our knowledge, no studies adding *B. velezensis* to silage in combination with other additives have been conducted to date.

In this study, we isolated and screened a strain identified as *B. velezensis* BV-10 with cellulose-degrading abilities from the termite gut. We sequenced the whole genome of *B. velezensis* BV-10 and annotated it using various databases to reveal its cellulose-degrading abilities. Then, we pretreated king grass silage with *B. velezensis* BV-10 and determined the changes in feed nutrient composition and fermentation quality with *B. velezensis* BV-10 alone and in conjunction with other silage additives to evaluate this *Bacillus* strain as a potential microbial additive for silage.

## 2. Materials and Methods

### 2.1. Chemicals and Preparation of Raw Materials

Carboxymethylcellulose sodium and Congo red were purchased from Boaotoda Technology Co., Ltd. (Beijing, China). Cellulase complex preparation (Ce) was purchased from Source Leaf Biotechnology Co., Ltd. (Shanghai, China). Molasses (Mo) was purchased from Jin Qian Wan Molasses Co., Ltd. (Liuzhou, China). A Gram-stain kit was purchased from Solarbio Technology Co., Ltd. (Beijing, China). All other chemicals were purchased from Xilong Scientific Technology Co., Ltd. (Guangzhou, China).

Samples of termites (*Coptotermes formosanus* species) were collected from Jinniu Ridge Park (Haikou, China). King grass was collected from Reyan No.4 King grass experimental field in Danzhou Tropical Agricultural Base (Danzhou, China). The termites were washed with saline and rinsed with 75% alcohol for approximately five minutes. After the alcohol was volatilized, the termite guts were removed and transferred to a sterile crucible, 1 mL of saline was added, and the gut samples were then ground thoroughly to obtain the termite intestinal abrasive solution.

### 2.2. Isolation of Cellulose-Degrading Bacteria from Termite Gut Samples

Termite intestinal abrasive solution was diluted and coated in a carboxymethylcellulose (CMC) primary screening medium (CMC-Na 10.0 g; K_2_HPO_4_ 4.0 g; (NH_4_)_2_SO_4_ 2.0 g; MgSO_4_·7H_2_O 0.3 g; peptone 2.0 g; agar 15.0 g in 1 L) to screen for cellulose-degrading bacteria. The coated primary-screened medium was transferred to a 37 °C constant-temperature incubator. After 10 h of culture, the samples were observed at 1-h intervals until distinct single colonies became visible. Each colony was marked according to its shape, size, smoothness, color, and other morphological characteristics. The single colonies were then inoculated into a new CMC primary screening medium to isolate pure bacteria. The pure colonies were rescreened with Congo red agar media (Congo red 0.4 g; CMC-Na 10.0 g; K_2_HPO4 4.0 g; (NH_4_)_2_SO_4_ 2.0 g; MgSO_4_·7H_2_O 0.3 g; peptone 2.0 g; agar 15.0 g in 1 L). The hydrolytic ring diameter D (mm) and colony diameter d (mm) were determined in Congo red agar media, and the D/d ratio was calculated to screen the strains with strong cellulose-degradation abilities.

### 2.3. 16S rDNA Identification

Isolated pure bacterial cultures were sent to Personal Biotechnology Co., Ltd. (Shanghai, China) for 16S rDNA identification. The sequences were compared to NCBI gene database BLAST sequences, and those with the highest similarity were downloaded. A phylogenetic tree was constructed using MEGA 7.0 software for analysis.

### 2.4. Whole-Genome Sequencing of B. velezensis BV-10

A single colony of *B. velezensis* BV-10 was picked, inoculated into Luria–Bertani (LB) liquid medium, and cultured in a 35 °C constant-temperature shaker at 180 rpm. When the OD600 of the bacterial solution reached 0.6, the bacterial cells were collected by centrifugation and washed by adding 1× phosphate buffered saline (PBS) 1 or 2 times until the supernatant was clarified and transparent. Then, the supernatant was removed and the bacterial cells were snap-frozen in liquid nitrogen for 15 min and transported to Personal Biotechnology Co., Ltd. (Shanghai, China) for whole-genome sequencing.

The genomic DNA of *B. velezensis* BV-10 was extracted using cetyltrimethylammonium bromide (CTAB), the total DNA content was measured using fluorescent dyes, and the DNA integrity was determined by 1% agarose gel electrophoresis. Libraries with different insertion fragments were constructed by the whole-genome shotgun method using second-generation sequencing technology and third-generation molecular sequencing technology based on the Illumina NovaSeq (Illumina Novaseq, San Diego, CA, USA) and PacBio Sequel (Pacific Biosciences, Menlo Park, CA, USA) sequencing platforms. AdapterRemoval [21] was used to remove splice contamination at the 3′ end, SOAPec [22] was used to quality correct all reads based on the Kmer frequency, and the Kmer used for quality correction was 17. The downlinked data obtained by Pacbio were spliced to obtain the contig sequence, which was corrected using Pilon 1.24 software [23]. Finally, the complete sequence was obtained by splicing.

Gene prediction covering the whole genome was performed using GeneMarkS 4.28 software [24]. The tRNAscan-SE [25], RNAmmer [26], and Rfam [27] functions were used to predict tRNA, rRNA, and ncRNA in the whole genome, and the protein-coding gene function was annotated by searching the Gene Ontology (GO) [28], Clusters of Orthologous Gene (COG) [29], and Kyoto Encyclopedia of Genes and Genomes (KEGG) [30] databases for the whole genome of *B. velezensis* BV-10. The Carbohydrate-Active enZYmes (CAZy) database [31] and the Virulence Factors Database (VFDB) of bacterial pathogens [32] were used to predict carbohydrate-activating enzymes and virulence factors, respectively, in the whole genome of *B. velezensis* BV-10. The genome-sequence, gene-prediction, and non-coding-RNA-prediction data were finally integrated into a standard GenBank-compatible format file, and the genome circle map was plotted using cgview [33].

### 2.5. Experimental Design of King Grass Silage

King grass at 8 weeks of growth was used as the raw material. King grass was placed in a ventilated area to dry overnight, chopped to a 2–3 cm length, mixed, and divided into 6 groups. The 6 groups were as follows: (1) control (CK; without additives), (2) molasses (MO), (3) cellulase (CE), (4) *B. velezensis* BV-10 (VEL), (5) VEL+MO, and (6) VEL+CE. The additives used in this experiment were 2.0% MO, 2.0% CE, and 1.0% VEL. VEL at 1.0% contained 6.7 × 10^5^ cfu/g fresh weight, 1.0% CE contained 10 U/g fresh weight, and 1.0% MO contained 10g/kg fresh weight. An equal amount of distilled water was added to each group. All the forages (approximately 180 g/group) were packed manually into pre-weighed polyethene plastic bags (20 × 30 cm) and sealed with a vacuum extractor [34], and each group contained three replicates. The silage was stored at 25–30 °C for 30 days. The sealed bags were opened for sampling and analysis on days 10, 20, and 30.

### 2.6. Quality Index and Nutritional Analysis of Silage

Silage samples (20 g) were homogenized in 70 mL of sterile distilled water and allowed to stand overnight at 4 °C. The samples were then filtered twice through filter paper, and the filtrate was used to determine the pH value and ammoniacal-nitrogen (AN) and organic-acid concentrations. The pH value was measured with a glass electrode pH meter (STARTER 3100, OHAUS International Trade Co., Ltd., Shanghai, China). The AN concentration was determined using a phenol-sodium hypochlorite colorimetric method [35]. The organic acids, including lactic acid (LA), acetic acid (AA), propionic acid (PA), and butyric acid (BA), were analyzed by high-performance liquid chromatography (Column: Venusil XBP C18(2), Agela, China; detector: SPD-M20A, Shimadzu Co., Ltd., Kyoto, Japan; mobile phase, 1 mmol/L of Na_2_HPO_4_; flow rate, 1 mL/min; temperature, 26 °C; injection volume, 10 µL; and SPD, 210 nm) [36].

The remaining silage samples were dried at 65 °C for 48 h to measure dry matter (DM), then pulverized and passed through a 40-mesh sieve for routine nutrient analysis. Kjeldahl nitrogen determination [37] was used for crude protein (CP) concentration, and Van Soset analysis [38] was used for neutral-detergent fiber (NDF) and acid-detergent fiber (ADF) concentration. The concentration of water-soluble carbohydrates (WSC) was determined by anthrone-concentrated-sulfuric acid colorimetry [39].

### 2.7. Statistical Analysis

The conventional nutrient composition and fermentation parameters of the silage were evaluated by two-way analysis of variance (ANOVA) using SPSS 23.0 software (SPSS Inc., Chicago, IL, USA). The data are expressed as means ± standard deviations. Graphs were prepared using GraphPad Prism 8.0.1 software. Multiple comparisons were performed using Duncan’s analysis, and *p* < 0.05 was considered to indicate significance.

## 3. Results

### 3.1. Isolation and Identification of B. velezensis BV-10

**Figure 1:**
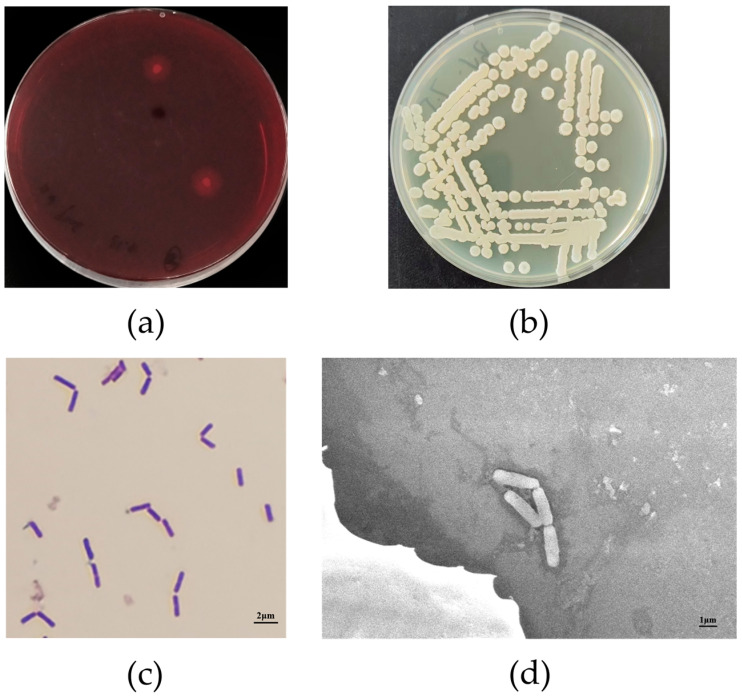
(**a**) Observation of hydrolyzed circles in Congo red agar medium (**b**) Colony morphology of the *Bacillus velezensis* BV-10 strain. (**c**) Gram staining of *B. velezensis* BV-10. (**d**) Microscopic morphology of *B. velezensis* BV-10.

**Figure 2:**
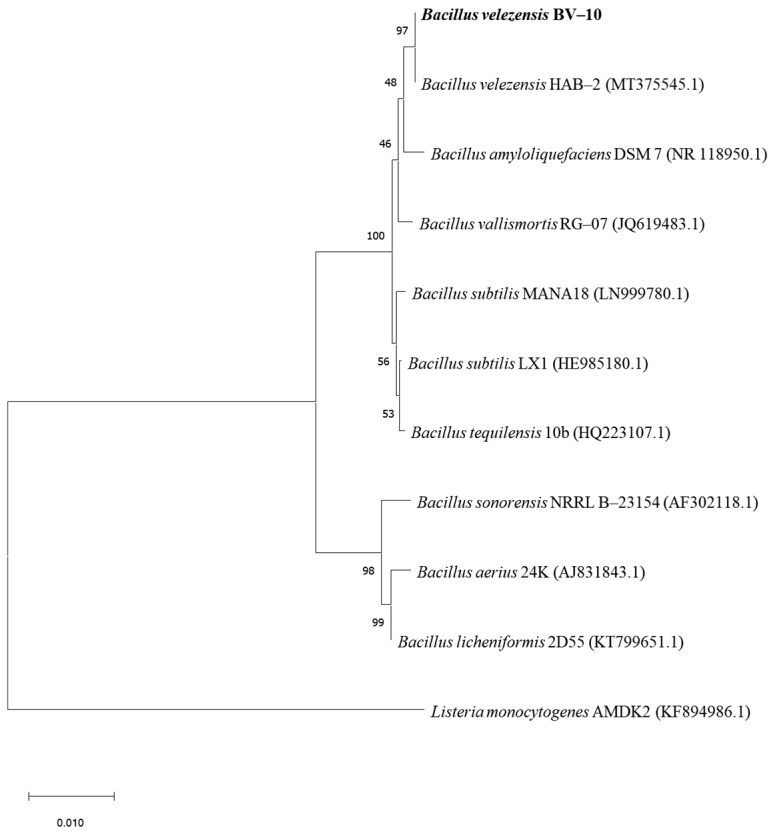
16S rDNA-sequence phylogenetic tree of *B. velezensis* BV-10.

Nineteen pure bacterial strains, including J1–J19, were isolated on CMC primary screening medium. Among the nineteen pure bacterial strains, J10 exhibited the highest cellulose-hydrolysis capacity, with the highest ratio of D/d (D: 18.0 mm, d: 3.3 mm, D/d: 5.45; Figure 1a). After incubation of J10 in LB solid medium at 37 °C for 24 h, the colonies were rounded and raised, with moist, yellowish colony surfaces. The colony surfaces wrinkled as the incubation time increased (Figure 1b). Gram staining and microscopic observation revealed the strain to be Gram-positive (Figure 1c). Scanning electron microscopy revealed that the strain was rod-shaped and approximately 0.8 × 2.0 μm in size (Figure 1d). Subsequently, the 16S rDNA sequence of the strain was similarity-matched using the BLAST function of NCBI and the phylogenetic tree was constructed (Figure 2). The strain was identified as *B. velezensis* and named *B. velezensis* BV-10. This strain has now been deposited in the Chinese Typical Cultures Depository Centre (Wuhan, China) under the depository number CCTCC No. M 2021357.

### 3.2. Whole-Genome Sequencing of B. velezensis BV-10 and Functional Annotations

**Figure 3:**
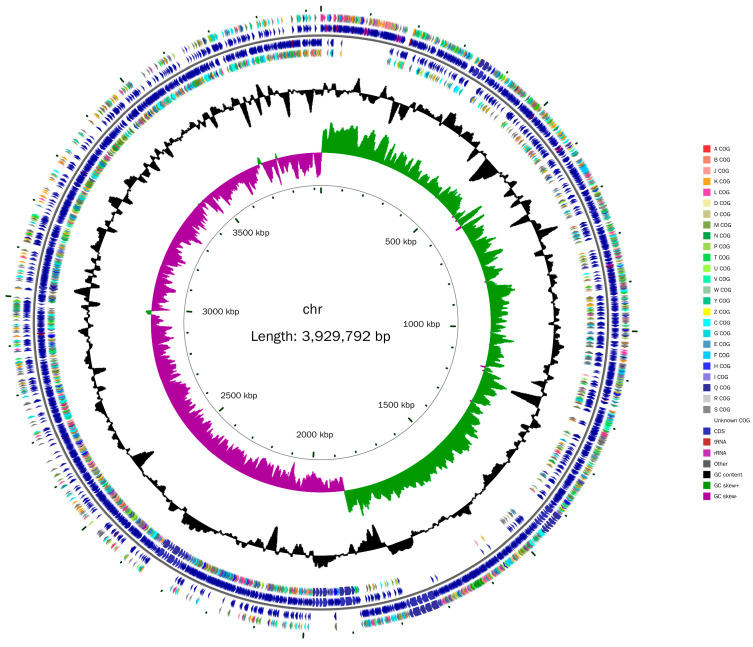
The complete genome of *B. velezensis* BV-10. From inside to outside, the first circle represents the scale; the second represents the GC skew; the third represents the GC content; the fourth and seventh represent the COG to which each CDS belongs; and the fifth and sixth represent the position of the CDS, tRNA, and rRNA on the genome.

The whole genome of *B. velezensis* BV-10 consists of a circular chromosome of 3929792 bp with 45.51% GC content (Figure 3) and contains 3873 protein coding genes (CDSs), 86 tRNAs, 27 rRNAs, and 82 ncRNAs (Tables S1 and S2). The GO, eggNOG, and KEGG databases revealed 2737, 3355, and 2163 protein-coding genes, respectively; other database information is provided in the Supplementary Material (Table S3).

**Figure 4:**
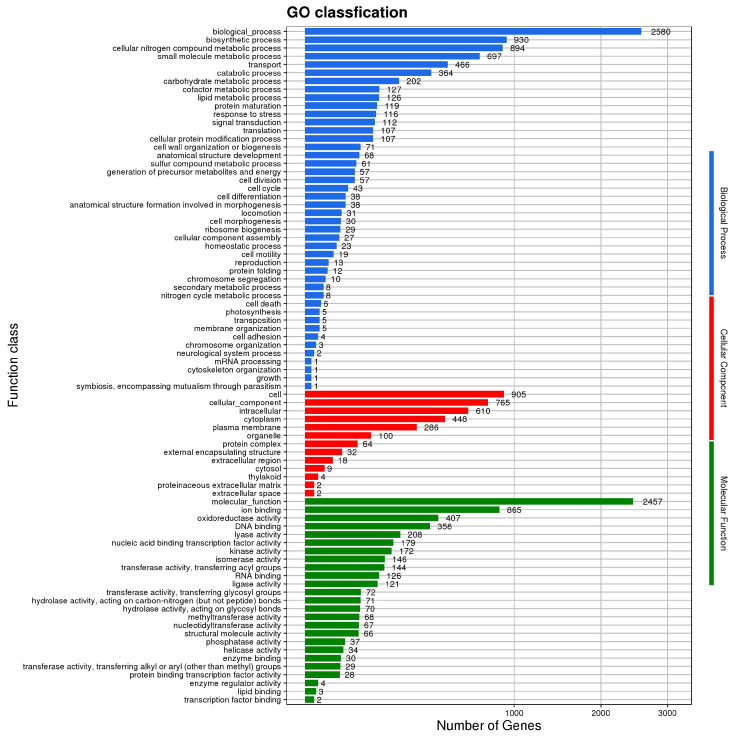
GO annotation of the *B. velezensis* BV-10 genome.

**Figure 5:**
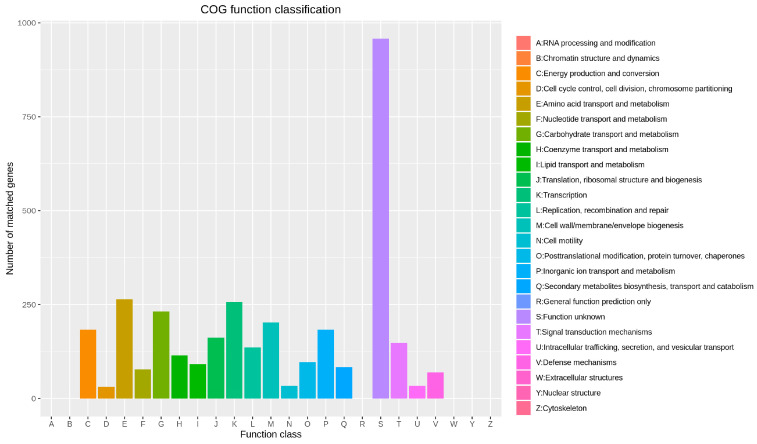
Diagram showing eggNOG functional classification of the *B. velezensis* BV-10 genome.

The GO database comprises three ontologies describing the gene’s molecular function, cellular location, and biological processes. The results of GO annotation for the *B. velezensis* BV-10 genome are shown in Figure 4; 7624 biological processes, 5764 molecular functions, and 3245 cellular-component annotations were obtained. In the eggNOG database annotation (Figure 5), *B. velezensis* BV-10 was found to have 3355 gene sequences divided into 20 types. Among these sequences, 958 had unknown functions (24.73%), requiring further research. Among the known functionally annotated g *B. velezensis* BV-10 genes, those involved in amino-acid transport and metabolism were the most numerous (264 genes; 6.82%), followed by those involved in transcription (257 genes); translation, ribosome structure and biogenesis (162 genes); carbohydrate transport and metabolism and lipid transport and metabolism (232 and 91 genes, respectively); and biosynthesis of secondary metabolites, transport and catabolism metabolism (83 genes).

**Figure 6:**
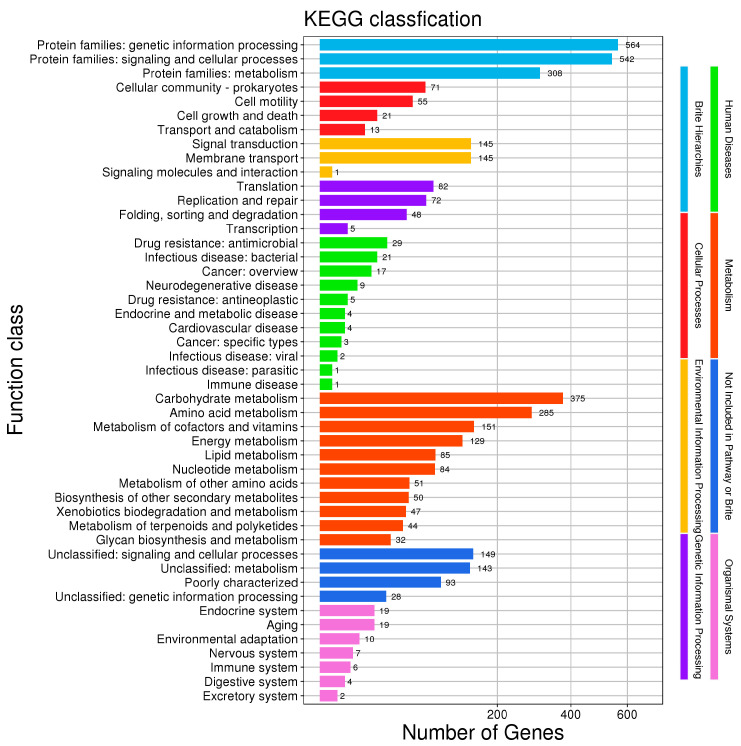
KEGG classification of the *B. velezensis* BV-10 genome.

A total of 2163 *B. velezensis* BV-10 genes were annotated in the KEGG database and classified into 8 major classes and 47 subclasses (Figure 6), of which the protein families genetic information processing, metabolism, signaling, and cellular processes contained the greatest number of genes, followed by carbohydrate metabolism and amino-acid metabolism.

### 3.3. Analysis of Carbohydrate-Active Enzymes (CAZymes) and Pathogenic Bacterial Virulence Factors

**Figure 7:**
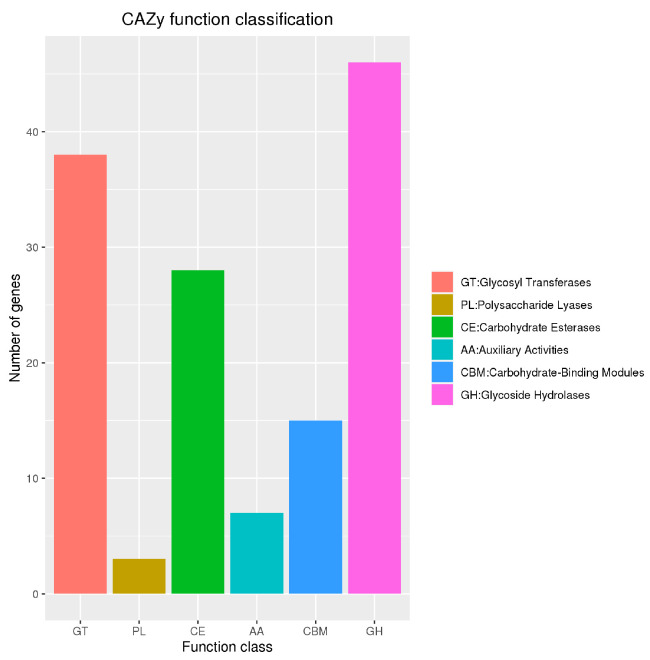
Numbers of genes belonging to carbohydrate-active enzyme families in *B. velezensis* BV-10.

A total of 137 *B. velezensis* BV-10 genes were annotated in the CAZy database (Figure 7); most (46 genes) were glycoside hydrolases, followed by glycosyltransferases (38 genes) and carbohydrate esterases (28 genes). Other detailed data are shown in Table S4. Lignocellulose-degrading enzymes encoded by *B. velezensis* BV-10 were also annotated, and the common genes are shown in Table 1. These genes are mainly classified as cellulose-degrading, hemicellulose-degrading, and lignin-degrading genes. Among them, GH3 encodes β-glucosidase, GH13 encodes a-amylase, GH11 encodes xylanase, and PL1 encodes pectin lyase. The results of VFDB database annotation the *B. velezensis* BV-10 genome are shown in Table S5, with a total of 20 genes annotated; no virulence factors detrimental to animal health were observed.

### 3.4. Effect of Different Additives and Silage Fermentation Time on the Conventional Nutrient Composition of King Grass during Silaging

Table 2 shows the contents of DM, CP, NDF, ADF, and WSC in silage treated with different additives at 10, 20, and 30 days of silage fermentation. The DM content of all groups did not change significantly with increasing silage time (*p* > 0.05). In addition, at 30 days, the CP content of the MO and VEL+MO groups was significantly lower than that at 10 days (*p* < 0.05) and the CP content of all other groups was significantly higher than that measured at 10 days of silage fermentation (*p* < 0.05). The CP content of the VEL and VEL+MO groups was significantly higher than that of the CK group (*p* < 0.05). The NDF and ADF content in both the VEL+MO and VEL+CE groups decreased with increasing silage fermentation time (*p* < 0.05). The NDF and ADF content in the VEL+MO group was the lowest among all groups at 30 days, followed by that in the VEL+CE group. The VEL group had significantly lower ADF content at 30 days of silage fermentation than did the CK group (*p* < 0.05). WSC content decreased significantly with increasing silage fermentation time in all groups (*p* < 0.05), and WSC levels in the MO and VEL+MO groups were significantly higher than those in all other groups at 10 and 30 days (*p* < 0.05). WSC content in the CE, VEL, and VEL+CE groups was significantly lower than that in the CK group (*p* < 0.05).

### 3.5. Effect of Different Additives and Silage Time on the Fermentation Quality of King Grass Silage

Table 3 shows the pH, LA, AA, PA, and AN concentrations in the silage fermentation broths from different additive treatments at 10, 20, and 30 days of silage fermentation. The pH value in all groups decreased significantly with increasing silage fermentation time (*p* < 0.05). The LA concentrations increased significantly with increasing silage fermentation time in all groups and were significantly higher in the VEL+MO group than in the CK group at 30 days (*p* < 0.05). At 10 days, the AA concentrations were significantly higher in the CE, VEL, VEL+MO, and VEL+CE groups than in the CK group (*p* < 0.05); however, at 30 days of silage fermentation, only the MO and CE groups had significantly higher AA concentrations than the CK group (*p* < 0.05). The PA concentrations of the VEL group were significantly lower than those in the VEL+CE group at day 30 (*p* < 0.05), and there were no significant differences in PA concentration among the other groups (*p* > 0.05). BA was not detected in any group at any time. The AN concentration in VEL+MO and VEL+CE groups increased and then decreased with increasing silage fermentation time (*p* < 0.05), and at 30 days, the AN content in the MO and VEL+MO groups was significantly higher than that in the CK group (*p* < 0.05).

## 4. Discussion

King grass is commonly used as feed for tropical ruminants because of its high dry-matter content [40]; however, its high content of lignocellulose, which is difficult to degrade, hinders the improvement of its nutritive value; thus, the nutritive value of silage is improved by adding cellulase [41] and microbial additives [42] to promote cellulose degradation. In this study, a bacterial strain with cellulose-degradation abilities was screened from wood-eating termites, identified as *B. velezensis*, and named *B. velezensis* BV-10. The whole genome of *B. velezensis* BV-10 was sequenced, and the strain was applied to king grass silage to explore its potential as a microbial additive.

In recent years, research on the cellulose-degradation ability of different sources of *B. velezensis* has increased. Chen et al. [43] isolated *B. velezensis* 157 from the bark of *Eucommia dulcis*, then sequenced its whole genome and found that *B. velezensis* 157 has genes encoding cellulose-, xylan-, lignin-, and starch-degrading enzymes. *B. velezensis* 157 was also applied to agro-industrial wastes in solid-state fermentation and to the production of industrial-valuable enzymes with industrial value. Similarly, we sequenced the whole genome of *B. velezensis* BV-10 and found that *B. velezensis* BV-10 has numerous genes encoding cellulose-, hemicellulose-, and lignin-degrading enzymes in the CAZy database, including β-glucosidase, xylanase, laccase, and amylase. These results were similar to the findings of Chen et al. [20]. Common genes from the GH1, GH3, GH4, GH5 and GH16 families participate in cellulose degradation. These genes include 6-phospho-β-glucosidase, β-glucosidase, 6-phospho-α-glucosidase, endo-1,4-β-glucanase and β-1,3(4)-glucanase. Endo-1,4-β-glucanase and β-glucosidase are two enzymes in the cellulase system [44]. GH30, GH43 and GH51 are also considered important components of hemicellulose degradation. GH43 is an important component of the xylan-degradation system [45]. Additionally, laccase, which belongs to AA1_2, was thought to play an important role in lignin degradation [46]. Laccases are multi-copper oxidases capable of single-electron oxidation of organic compounds to free radicals. Laccase enables other enzyme systems to access cellulose and hemicellulose by modifying the properties of lignin [47]. These enzymes are collectively involved in the degradation of lignocellulose.

*B. velezensis* was presumed to be a candidate for the QPS list due to its “absence of toxin production potential” and “absence of aminoglycoside production or genes encoding aminoglycosides” [48]. Khalid et al. [49] reported that *B. velezensis* has beneficial effects on animal growth and has substantial potential as a probiotic in animal feed. Regarding antimicrobials, *B. velezensis* uses 10% of its genome to synthesize antimicrobial molecules that inhibit phytopathogenic bacteria and has been used as a biocontrol agent for many plant diseases [50,51]. The use of *B. velezensis* in king grass silage has not been fully explored. Therefore, we added a set concentration of *B. velezensis* BV-10 to king grass silage and examined the changes in the conventional nutrients and fermentation quality of the silage over 30 days. DM is related to factors such as silage packing density and fermentation pattern [52]. In this experiment, the DM content did not decrease significantly over time, indicating that the technique resulted in good preservation of the king grass. CP is a factor in evaluating the feed’s nutritive value; microbial activity decreases the CP content [53]. With the addition of *B. velezensis* BV-10, the CP content was still high at 30 days of silage. This effect was probably due to the antimicrobial activity of *B. velezensis*, which inhibits the proliferation of spoilage bacteria in the silage, thus reducing protein degradation [51,54]. However, microbial activity still resulted in a decrease in the CP content as the silage fermentation time increased, and the accumulation of AN could also indicate that CP was continuously consumed during ensiling. Chen et al. [55] isolated *B. velezensis* CL-4 from the chicken cecum and applied it to NaHCO_3_-treated maize germ meal, revealing that the bacterial strain promoted lignocellulose degradation. Uddin et al. [56] showed that increasing the rate of crude-fiber degradation in silage is beneficial for increasing the rate of feed intake by ruminants, thereby improving production performance. In this study, when *B. velezensis* BV-10 was added to silage mixed with MO or CE, the NDF and ADF content was significantly reduced, indicating that lignocellulose in silage was degraded, whereas the degradation of NDF and ADF was not significant when *B. velezensis* BV-10 and MO were added alone; thus, it was hypothesized that the addition of BV10 in conjunction with MO increased the cellulose-degradation capacity of *B. velezensis* BV-10 and the degradation of NDF and ADF in the silage at 30 days. The degradation effect on NDF and ADF exceeded that of CE at this time point. WSC is the energy source for microbial proliferation and growth, and the WSC content and activity of natural lactic acid bacteria together determine the rate of pH decline at the early stage of silage [57]. The pH value of silage is an essential index for evaluating its fermentation quality. A pH value below 4.2 is considered to indicate well-fermented silage [58]. A rapid decrease in pH value during the early stages of silage inhibits the production of harmful microorganisms and improves the feed fermentation quality [59]. In this study, the addition of *B. velezensis* BV-10 resulted in a rapid decrease in the pH value of the silage; when *B. velezensis* BV-10 and MO were added to the silage at the same time, the WSC content of the feed was high at the beginning of the silage period and the pH value decreased the most from 10 to 20 days of silage, reaching 3.77 at 20 days. This value was significantly lower than that in the control group. The addition of MO increased the level of WSC in the silage, and increasing the level of WSC could reduce the pH value of the feed during 30 days of silage [60]. This effect might explain the rapid decrease in the pH value of the silage that followed the addition of MO. The pH value of all treatments was below 4.2 at 30 days, which meets the criterion for good-quality silage fermentation.

Rapid acidification of silage is closely related to LA production, which is necessary to prevent early colonization by undesirable microorganisms and nutrient loss [61]. The king grass silage supplemented with *B. velezensis* BV-10 and MO accumulated the most LA content at 30 days, followed by the treatment group supplemented with *B. velezensis* BV-10 and CE; both groups also showed the fastest decrease in pH, probably because the simultaneous addition of *B. velezensis* BV-10 and MO can inhibit the propagation of undesirable microorganisms in silage and provide excellent conditions for lactic acid bacteria fermentation. AA is an essential preservative in tropical forage silage [62]. When the WSC content of silage is low, the lactic acid bacteria in silage will turn to heterofermentation to produce AA [63], and the production of AA was reduced because the treatment group with *B. velezensis* BV-10 and MO added had a higher WSC content. In this study, PA accumulated in silage over time, and the treatment group with *B. velezensis* BV-10 and CE added had the greatest accumulation of PA at 30 days, potentially due to the presence of higher levels of *Propionibacterium* [64].

## 5. Conclusions

This study found that *B. velezensis* BV-10 isolated from termite intestine possesses genes encoding various lignocellulases and that this bacterial strain can promote cellulose degradation in king grass silage, rapidly decrease the pH value of the silage, and improve the quality of silage fermentation. When mixed and added in conjunction with MO or CE, *B. velezensis* BV-10 can decrease NDF and ADF in silage, decrease the consumption of CP in silage, and promote the accumulation of organic acids in feed. *B. velezensis* BV-10 has the best effect when it is added in combination with MO.

## Figures and Tables

**Table 1:** Annotated genes encoding lignocellulose-degrading enzymes in *B. velezensis* BV-10.

Classification	CAZy	Number of ORFs Annotated	Enzymes
Cellulose-related	GH1	2	6-phospho-β-glucosidase (EC 3.2.1.86)
	GH3	1	β-glucosidase (EC 3.2.1.21)
	GH4	3	6-phospho-β-glucosidase (EC 3.2.1.86) 6-phospho-α-glucosidase (EC 3.2.1.122)
	GH5	2	endo-1,4-β-glucanase (EC 3.2.1.4)
	GH13	6	a-glucosidase (EC 3.2.1.20) a-amylase (EC 3.2.1.1)
	GH16	1	β-1,3(4)-glucanase (EC 3.2.1.6)
	GH32	3	endo-levanase (EC 3.2.1.65) sucrose-6-phosphate hydrolase (EC 2.4.1.–)
	PL1	2	pectin lyase (EC 4.2.2.10) pectate lyase (EC 4.2.2.2)
	PL9	1	pectate lyase (EC 4.2.2.2)
Hemicellulose-related	GH11	1	xylanase (EC 3.2.1.8)
	GH26	1	β-mannanase (EC 3.2.1.78)
	GH30	2	glucosylceramidase (EC 3.2.1.45)
	GH43	3	arabinan endo-1,5-α-L-arabinosidase (EC 3.2.1.99) arabinoxylan arabinofuranohydrolase (EC 3.2.1.–) 1,4-β-xylosidase (EC 3.2.1.37)
	GH51	2	α-L-arabinofuranosidase (EC 3.2.1.55)
	CE3	1	acetyl xylan esterase (EC 3.1.1.72)
Lignin-related	AA1_2	1	laccase (EC 1.10.3.2)
	AA4	1	vanillyl-alcohol oxidase (EC 1.1.3.38)
	AA6	1	1,4-benzoquinone reductases (EC 1.6.5.6)
	AA7	3	FAD-binding protein (EC 1.1.3.–) FAD-dependent oxidase (EC 1.1.3.–)
	AA10	1	copper-dependent lytic polysaccharide monooxygenases (EC 1.14.99.53)

**Table 2:** Changes in the conventional nutrient composition of king grass during ensiling.

Item	Ensiling Days	Treatment					
		CK	MO	CE	VEL	VEL+MO	VEL+CE
DM (%FM)	10	25.55 *±* 1.05 ^a^	24.54 *±* 0.29 ^ab^	23.52 *±* 1.14 ^b^	24.57 *±* 0.97 ^ab^	23.64 *±* 0.78 ^ab^	23.27 *±* 1.45 ^b^
	20	25.06 *±* 0.40	24.57 *±* 0.56	23.65 *±* 1.15	23.87 *±* 0.62	24.55 *±* 1.44	24.02 *±* 0.82
	30	24.07 *±* 1.60	23.81 *±* 0.86	24.72 *±* 1.23	24.30 *±* 0.80	24.18 *±* 1.09	23.94 *±* 2.24
CP (%DM)	10	5.61 *±* 0.12 ^b^	5.81 *±* 0.06 ^bA^	5.25 *±* 0.05 ^cB^	5.76 *±* 0.20 ^bB^	7.01 *±* 0.19 ^aA^	5.12 *±* 0.15 ^cB^
	20	5.53 *±* 0.11 ^d^	5.81 *±* 0.26 ^cdA^	5.07 *±* 0.08 ^eB^	6.35 *±* 0.14 ^bA^	6.72 *±* 0.13 ^aAB^	5.88 *±* 0.18 ^cA^
	30	5.77 *±* 0.13 ^c^	5.43 *±* 0.12 ^dB^	5.86 *±* 0.17 ^cA^	6.20 *±* 0.13 ^bA^	6.57 *±* 0.22 ^aB^	6.03 *±* 0.10 ^bcA^
NDF (%DM)	10	76.60 *±* 1.56 ^a^	73.97 *±* 0.95 ^bB^	75.03 *±* 1.33 ^abAB^	76.07 *±* 0.91 ^abB^	74.63 *±* 1.86 ^abA^	75.10 *±* 0.52 ^abA^
	20	78.80 *±* 1.83 ^a^	76.17 *±* 0.59 ^abA^	76.63 *±* 1.55 ^abA^	78.57 *±* 1.17 ^aA^	74.17 *±* 1.79 ^bA^	76.23 *±* 2.18 ^abA^
	30	76.9 *±* 0.96 ^a^	74.2 *±* 0.66 ^bB^	73.53 *±* 0.93 ^bB^	76.13 *±* 0.59 ^aB^	70.27 *±* 0.21 ^cB^	71.37 *±* 1.27 ^cB^
ADF (%DM)	10	50.20 *±* 0.40 ^bB^	49.00 *±* 0.10 ^bB^	49.83 *±* 1.19 ^bB^	49.30 *±* 0.82 ^bB^	49.40 *±* 0.44 ^bA^	52.63 *±* 2.46 ^aA^
	20	50.53 *±* 0.68 ^bcAB^	51.17 *±* 0.49 ^bA^	53.47 *±* 0.40 ^aA^	50.70 *±* 0.60 ^bcA^	49.13 *±* 1.62 ^cAB^	50.57 *±* 1.17 ^bcAB^
	30	51.37 *±* 0.38 ^aA^	50.80 *±* 0.79 ^aA^	50.83 *±* 0.32 ^aB^	48.80 *±* 0.66 ^bB^	47.13 *±* 0.81 ^cB^	48.17 *±* 1.24 ^bcB^
WSC (%DM)	10	14.24 *±* 0.77 ^bA^	18.41 *±* 0.43 ^aA^	14.27 *±* 0.39 ^bA^	12.96 *±* 0.60 ^bA^	17.64 *±* 0.92 ^aA^	13.31 *±* 0.89 ^bA^
	20	10.92 *±* 0.24 ^bB^	12.13 *±* 0.29 ^aB^	10.62 *±* 0.61 ^bB^	10.30 *±* 0.35 ^bB^	10.88 *±* 0.90 ^bB^	9.95 *±* 0.47 ^bB^
	30	9.33 *±* 0.63 ^abC^	10.19 *±* 0.32 ^aC^	8.22 *±* 0.36 ^cC^	8.28 *±* 0.24 ^cC^	8.94 *±* 0.35 ^bcC^	8.46 *±* 0.89 ^bcB^

DM, dry matter; FM, fresh matter; CP, crude protein; NDF, neutral detergent fiber; ADF, acid detergent fiber; and WSC, water-soluble carbohydrates. Data are means of three samples; means with different letters in the same row (a–e) or column (A–C) differ significantly (*p* < 0.05).

**Table 3:** Changes in the fermentation quality of king grass silage during ensiling.

Item	Ensiling Days	Treatment					
		CK	MO	CE	VEL	VEL+MO	VEL+CE
pH	10	4.61 *±* 0.17 ^aA^	4.43 *±* 0.04 ^abA^	4.50 *±* 0.09 ^abA^	4.40 *±* 0.06 ^bA^	4.34 *±* 0.14 ^bcA^	4.17 *±* 0.03 ^cA^
	20	4.40 *±* 0.27 ^aAB^	4.21 *±* 0.14 ^abB^	4.07 *±* 0.13 ^bcB^	4.03 *±* 0.11 ^bcB^	3.77 *±* 0.19 ^bB^	3.91 *±* 0.04 ^bcB^
	30	4.07 *±* 0.06 ^aB^	4.06 *±* 0.06 ^aB^	3.88 *±* 0.11 ^bB^	4.02 *±* 0.04 ^abB^	3.98 *±* 0.10 ^abB^	3.64 *±* 0.04 ^cC^
LA (mg/mL)	10	15.41 *±* 0.82 ^aC^	14.45 *±* 1.09 ^abB^	11.39 *±* 1.52 ^cC^	13.07 *±* 1.63 ^bcC^	15.62 *±* 0.60 ^aC^	14.14 *±* 0.28 ^abB^
	20	17.75 *±* 1.33 ^abB^	13.44 *±* 0.51 ^cB^	15.08 *±* 1.05 ^bcB^	17.30 *±* 1.72 ^abB^	20.25 *±* 1.13 ^aB^	17.98 *±* 3.35 ^abB^
	30	20.98 *±* 0.57 ^bcA^	19.39 *±* 1.52 ^cA^	22.29 *±* 2.58 ^bcA^	20.59 *±* 0.95 ^bcA^	27.92 *±* 2.04 ^aA^	23.21 *±* 2.62 ^bA^
AA (mg/mL)	10	8.26 *±* 1.54 ^cB^	10.22 *±* 1.04 ^bcB^	11.96 *±* 1.68 ^abB^	12.86 *±* 0.91 ^aA^	12.15 *±* 0.39 ^abB^	12.69 *±* 0.40 ^a^
	20	9.32 *±* 2.30 ^cB^	11.85 *±* 1.93 ^bcB^	10.64 *±* 1.62 ^cB^	10.09 *±* 0.29 ^cB^	15.07 *±* 1.23 ^aA^	13.47 *±* 0.63 ^ab^
	30	14.22 *±* 0.21 ^cA^	17.80 *±* 1.22 ^abA^	19.04 *±* 1.09 ^aA^	13.25 *±* 0.42 ^cA^	15.13 *±* 1.45 ^bcA^	12.43 *±* 3.16 ^c^
PA (mg/mL)	10	11.68 *±* 1.05 ^aB^	9.25 *±* 1.15 ^bB^	10.71 *±* 0.64 ^abB^	11.14 *±* 1.64 ^abB^	9.31 *±* 0.43 ^aB^	11.01 *±* 0.27 ^abC^
	20	14.86 *±* 1.30 ^abA^	12.70 *±* 1.25 ^cA^	12.92 *±* 1.13b ^cAB^	11.04 *±* 0.88 ^cB^	16.43 *±* 1.11 ^aA^	14.88 *±* 1.06 ^abB^
	30	14.75 *±* 0.41 ^abA^	13.69 *±* 0.45 ^abA^	15.87 *±* 3.95 ^abA^	13.51 *±* 0.32 ^bA^	14.98 *±* 1.42 ^abA^	17.09 *±* 0.57 ^aA^
AN (mg/mL)	10	0.17 *±* 0.01 ^a^	0.18 *±* 0.03 ^a^	0.13 *±* 0.02 ^bB^	0.18 *±* 0.03 ^a^	0.17 *±* 0.01 ^aB^	0.15 *±* 0.01 ^bB^
	20	0.18 *±* 0.02 ^a^	0.19 *±* 0.01 ^a^	0.15 *±* 0.01 ^bA^	0.15 *±* 0.01 ^b^	0.19 *±* 0.01 ^aA^	0.18 *±* 0.02 ^aA^
	30	0.16 *±* 0.01 ^c^	0.19 *±* 0.01 ^a^	0.15 *±* 0.01 ^cA^	0.15 *±* 0.01 ^c^	0.18 *±* 0.01 ^bB^	0.15 *±* 0.01 ^cB^

LA, lactic acid; AA, acetic acid; PA, propionic acid; BA, butyric acid; AN, ammonia nitrogen; and ND, not detected. Data are means of three samples; means with different letters in the same row (a–c) or column (A–C) differ significantly (*p* < 0.05).

## Data Availability

Data are contained within the article.

## Supplementary Materials

The following supporting information can be downloaded at: https://www.mdpi.com/article/10.3390/microorganisms11112697/s1, Table S1: Statistics of open reading frame (OFR) predictions; Table S2: Statistics of non-coding RNA predictions; Table S3: Functional annotation of protein-coding genes; Table S4: Carbohydrate-active enzymes analysis statistics; Table S5: Virulence factors database analysis statistics.
