# Supplementary material for: Whole-Genome Analysis of Termite-Derived Bacillus velezensis BV-10 and Its Application in King Grass Silage
Source: Microorganisms. 2023 Nov 3;11(11):2697. doi: 10.3390/microorganisms11112697 (PMC10672971; doi:10.3390/microorganisms11112697)
Supplement: Supplementary file 1 [file microorganisms-11-02697-s001.zip › Supplementary Materials.pdf]

Table S1 Statistics of open reading frame (OFR) predictions

Property	Value
ORF num	3873
ORF total length	3481344 bp
ORF density	0.986 genes per kb
Longest ORF length	16173 bp
ORF average length	898.88 bp
Intergenetic region length	448448 bp
ORF/Genome(coding percentage)	88.59%
Intergenetic length/Genome	11.41%
GC content in ORF region	47.34%
GC content in intergenetic region	39.96%

Table S2 Statistics of non-coding RNA predictions

Type	Copy Number	Avg. length (bp)	Total length (bp)	percent of genome (%)
5S rRNA	9	111	999	0.0254
16S rRNA	9	1547	13923	0.3543
23S rRNA	9	2926	26334	0.6701
tRNA	86	77	6639	0.1689
ncRNA	82	152	12467	0.3172

Table S3 Functional annotation of protein-coding genes

Annotation in Database	Number of Genes	Percentage (%)
NR	3859	99.6385
eggNOG	3355	86.6254
KEGG	2163	55.8482
Swiss-Prot	3533	91.2213
GO	2737	70.6683

Table S4 Carbohydrate-active enzymes analysis statistics

Type	Number of Genes	Percentage (%)
Glycosyl Transferases	38	0.98
Polysaccharide Lyases	3	0.08
Carbohydrate Esterases	28	0.72
Auxiliary Activities	7	0.18
Carbohydrate-Binding Modules	15	0.39
Glycoside Hydrolases	46	1.19

Table S5 Virulence factors database analysis statistics

VFDB ID	Genes IDs	VFDB name
VFG000079(gb|NP_463763)	chr_96	VF0072
VFG046465(gb|WP_003028672)	chr_122	VF0460
VFG012095(gb|WP_003435012)	chr_602	VF0594
VFG002158(gb|NP_464456)	chr_993	VF0347
VFG000080(gb|NP_464522)	chr_1335	VF0073
VFG011430(gb|WP_002963616)	chr_1562	VF0367
VFG001259(gb|NP_250137)	chr_1606	VF0273
VFG048830(gb|WP_014907233)	chr_2220	VF0560
VFG050037(gb|WP_001133943)	chr_2921	VF0586
VFG050015(gb|WP_000955350)	chr_2923	VF0586
VFG049993(gb|WP_001100504)	chr_2925	VF0586
VFG045303(gb|WP_010946492)	chr_3190	VF0171
VFG000077(gb|NP_465991)	chr_3220	VF0074
VFG001373(gb|WP_000758382)	chr_3325	VF0144
VFG000681(gb|AAF13662)	chr_3347	VF0141
VFG000682(gb|AAF13663)	chr_3348	VF0141
VFG000270(gb|WP_000724295)	chr_3429	VF0050
VFG013286(gb|WP_005694325)	chr_3663	VF0044
VFG046645(gb|WP_003020694)	chr_3663	VF0543
VFG038144(gb|WP_001062908)	chr_3663	VF0465
